# Longitudinal relations between bullying victimization and aggression: The multiple mediation effects of anger rumination and hostile automatic thoughts

**DOI:** 10.1002/pchj.760

**Published:** 2024-04-24

**Authors:** Fangying Quan, Jianjian Huang, Honghan Li, Wenfeng Zhu

**Affiliations:** ^1^ Department of Psychology, Faculty of Education Guangxi Normal University Guilin China; ^2^ Guangxi University and College Key Laboratory of Cognitive Neuroscience and Applied Psychology, Faculty of Education Guangxi Normal University Guilin China; ^3^ Key Research Base of Humanities and Social Sciences of the Ministry of Education, Academy of Psychology and Behavior Tianjin Normal University Tianjin China

**Keywords:** aggression, anger rumination, bullying victimization, hostile automatic thoughts

## Abstract

Bullying victimization is widely accepted to be associated with aggression. However, the mechanisms underlying this relationship remain unknown. To examine the long‐term impact of bullying victimization on aggression, the present study tested the potential mediating effects of both anger rumination and hostile automatic thoughts. A total of 809 undergraduates from four universities across China (74.80% female; *M*_age_ = 19.63 years, *SD* = 0.82 years) completed the survey on three occasions, with a 6‐month delay between Time 1 and Time 2 and a 1‐year interval between Time 2 and Time 3. A cross‐lagged model of anger rumination and hostile automatic thoughts was developed to test whether they predicted one another, and two structural models were constructed to test their mediating roles in bullying victimization and aggression. Findings indicated that anger rumination and hostile automatic thoughts are mutually predictable; the correlation between bullying victimization and aggression is mediated independently by anger rumination and hostile automatic thoughts, and the chain mediation of bullying victimization predicting aggression first through anger rumination and then through hostile automatic thinking was established. In addition, an alternative mediation model is also significant, with hostile automatic thoughts as the primary mediator and anger rumination as the secondary mediator. These results highlight the significance of anger rumination and hostile automatic thoughts in the long‐term effects of bullying victimization on aggression. Interventions designed to reduce undergraduate students' anger rumination and hostile automatic thoughts may help reduce their aggression.

## INTRODUCTION

Bullying victimization, also known as peer victimization, is the process by which an individual is frequently exposed to intentional negative behaviors from peers, such as verbal bullying, physical bullying, social bullying, relationship damage, and threatening behaviors (Hamburger et al., 2011; Olweus, 1996; Shaw et al., 2013). Bullying is widely acknowledged as a serious issue in elementary and secondary schools, and it has been linked to a number of psychosocial and behavioral issues in both bullies and victims, including psychological distress (Peltzer & Pengpid, 2020), depression (Yang et al., 2022), anger rumination (Malamut & Salmivalli, 2021), perpetration (Walters, 2021), aggression (Ostrov et al., 2022), and suicidal ideation (X. Zhu et al., 2022). Additionally, those who are victims and perpetrators of violence have the highest rates of eating problems (Copeland et al., 2015; Kwan et al., 2017), maladjustment (Yang et al., 2016), and suicidality (Serafini et al., 2023). Recent evidence suggests that bullying victimization also occurs among college students (Lund & Ross, 2017; Pörhölä et al., 2020), and the consequences of bullying victimization for college students are just as severe as for elementary and secondary school students (Kwan et al., 2017; Víllora et al., 2020; M. C. Zhang et al., 2023). Lund and Ross (2017) discovered an average frequency of victimization among university students of between 20% and 25% after reviewing 14 studies. Comparably, a cross‐cultural study conducted at 47 colleges across four nations discovered that the incidence of bullying victimization among students ranged from 2.2% to 25.2%, indicating that a sizable portion of students encounter bullying from their peers while enrolled in school (Pörhölä et al., 2020). Another study that examined bullying victimization experiences among undergraduate students during the previous 3 months discovered that 16.9% of the participants had experienced three or four different types of bullying victimization (physical, verbal, social, or cyberbullying), and the victims also had the lowest stress resilience and the worst subjective well‐being (Víllora et al., 2020). Given that bullying also occurs frequently among college students, it is critical to study the impact of bullying victimization on psychosocial and behavioral problems among college students, as it can help to develop targeted prevention and intervention programs to improve college students' mental and physical health.

### Bullying victimization and aggression

Aggression is typically defined as behaviors that are directed towards another person with the direct intent of causing harm (Anderson & Bushman, 2002). The general aggression model (GAM; Allen et al., 2018) is a comprehensive paradigm for the understanding of aggressive behaviors in humans. GAM posits that environmental stressors, such as bullying victimization, can promote the formation of aggressive knowledge structures (e.g., aggressive behavioral scripts). These structures can change a person's aggressive personality and raise the possibility that they will act aggressively. Previous studies have demonstrated that being bullied is an important indicator of future aggressive behaviors (Cooley et al., 2018; Li et al., 2022; Nickerson et al., 2020; Ostrov et al., 2022; Walters & Espelage, 2023; Wang et al., 2022; Xiong et al., 2022; J. Zhang et al., 2022; M. C. Zhang et al., 2023). For example, in a study using longitudinal 3‐wave data with 198 elementary school students, Cooley et al. (2018) found that reports of peer victimization were able to predict levels of aggression both within and across an academic year. In a similar vein, a longitudinal study with 609 Chinese preadolescents found a strong correlation between experiencing school bullying and engaging in reactive and proactive aggression 1 year later (Li et al., 2022). Similar findings were made by J. Zhang et al. (2022), who discovered that bullying victimization positively and significantly predicts the online aggression of college students a year later.

Despite the widespread acceptance of the connection between bullying victimization and aggression, the question of how bully victims become aggressors remains open. According to the social‐ecological diathesis‐stress model (Swearer & Hymel, 2015), being bullied is a stressful life experience for individuals, and it can trigger cognitive vulnerabilities, which consist of characteristic possession of cognitive patterns or biased beliefs, such as hostility towards others. Once activated, cognitive vulnerabilities can cause a change in individuals' characteristic ways of organizing, attending to, mentally representing, interpreting, and remembering negative life events (Elwood et al., 2009) and then lead to more severe consequences (e.g., aggression, depression). Researchers have found that bullying victimization may lead to two typical cognitive vulnerabilities: anger rumination (Malamut & Salmivalli, 2021; Spyropoulou & Giovazolias, 2022) and hostile automatic thoughts (Edalati et al., 2018), which have been shown to be risk factors for aggressive behaviors in college students (Eisenlohr‐Moul et al., 2016; W. Zhu et al., 2023). A recent study found that being victimized by peers impacted college students' cognition negatively and ultimately led to aggressive behaviors (M. C. Zhang et al., 2023). However, few studies have explored the potential multiple mediating roles of both anger rumination and hostile automatic thoughts in the relationship between bullying victimization and aggression, and even fewer have involved a college‐age sample. Research on these issues can further our understanding of the causes of aggression and inform more effective methods of prevention and intervention on college campuses.

### The mediating role of anger rumination

Anger rumination refers to the repeated reflection on one's experience of anger, with a focus on the cause, process, and outcome of the event that triggered the anger, and can be accompanied by feelings of anger as well as thoughts of vengeance (Denson, 2013; Sukhodolsky et al., 2001). The social‐ecological diathesis‐stress model posits that the emergence of externalizing behavior problems is affected not only by environmental risk but also by activation of cognitive vulnerability (Elwood et al., 2009; Swearer & Hymel, 2015), and anger rumination has been identified as a cognitive vulnerability that may result in aggression (Harmon et al., 2019). Thus, we speculate that anger rumination as a cognitive vulnerability might mediate the relationship between bullying victimization and aggression. For example, one longitudinal study found that bullying victimization in the third and fourth grades can predict anger rumination about prior victimization experiences in the seventh grade (Malamut & Salmivalli, 2021). Another study found that peer victimization significantly predicted anger rumination 1 year later (Spyropoulou & Giovazolias, 2022). As a result, we believe that being the victim of bullying may increase the likelihood of ruminating on anger.

The multiple‐systems model of angry rumination posits that anger rumination may temporarily weaken one's self‐control, making it more difficult for the individual to suppress thoughts related to anger and shift attention, thus increasing the likelihood of aggression (Denson, 2013). For example, Pedersen et al. (2011) found that rumination following an anger‐inducing event alters people's internal state, increasing their level of rage, aggressive thoughts, and physiological arousal. This affects how they assess and make decisions regarding the anger‐inducing event, which in turn increases the likelihood that they will react aggressively. Evidence from diary studies (Eisenlohr‐Moul et al., 2016), experimental studies (Bushman et al., 2005), and longitudinal studies (Camacho et al., 2021; Harmon et al., 2019) further supports the notion that anger rumination can trigger aggressive behavior. With this in mind, we propose that the impact of bullying victimization on aggression may be mediated by anger rumination.

### The mediating role of hostile automatic thoughts

Hostile automatic thoughts refer to the automatic and recurring thoughts in the mind designed to physically assault, degrade, and retaliate against other people (Snyder et al., 1997). Beck's cognitive theory posits that when people encounter a specific situation, they automatically and rapidly recall ideas in both verbal and visual forms, which is known as automatic thoughts (Beck, 1976). Automatic thoughts can be either positive or negative; negative automatic thoughts include those related to physical threat, social threat, failure, and hostility (Hogendoorn et al., 2010), and these negative thoughts can influence a person's behavior. As there is still relatively little research on hostile automatic thoughts, we decided to focus on this facet in order to investigate its possible contribution to the influence of bullying victimization on aggression. Prior studies have found an association between bullying victimization and hostile‐related automatic thoughts (Edalati et al., 2018), as well as between hostile automatic thoughts and aggressive behaviors (Revill et al., 2020; W. Zhu et al., 2023). For instance, Edalati et al. (2018) found that adolescent peer victimization was associated with hostile automatic thoughts 4 years later, and Revill et al. (2020) discovered that hostile automatic thoughts were predictive of the emergence of adolescent externalizing behaviors, such as aggressive, hyperactive, and delinquent behaviors. Moreover, W. Zhu et al. (2023) found that hostile automatic thoughts predicted cyberaggression among college students 6 months later. We thus hypothesized that hostile automatic thoughts might mediate the relationship between bullying victimization and aggression.

### The relationship between anger rumination and hostile automatic thoughts

The social information processing (SIP; Crick & Dodge, 1994) model proposes that mental representations of prior experiences are retained in long‐term memory and can become integrated into a general mental structure, such as schema and script (Shank & Abelson, 1977), which then guides the processing of upcoming social cues. The SIP model suggests that aggression‐related cognitive factors may predict each other in cycles due to aggressive scripts or aggressive schema (Crick & Dodge, 1994; Huesmann & Eron, 1984). It means that certain aggressive cognitive factors (such as anger rumination) and other aggressive cognitive factors (such as hostile automatic thoughts) may affect each other. A previous study has shown that hostile attribution bias can significantly predict anger rumination after 6 months, and rumination has marginal significance in predicting hostile attribution bias across time (Wang et al., 2019). Anger rumination and hostile automatic thoughts can make individuals more likely to evoke scripts related to anger and hostility, which involve detailed recall of anger events (Wilkowski & Robinson, 2008), and the arousal of these anger or hostility scripts further promotes the generation and development of anger rumination and hostile automatic thoughts. We, therefore, assume that there are reciprocal relationships between anger rumination and hostile automatic thoughts. For example, in a study of adults with problematic anger, it was found that rumination can lead to excessive preoccupation with perceived erroneous behavior and theoretically exacerbate hostile automatic thoughts (Matheny et al., 2017). Similar to this, another study discovered that those who have a tendency to dwell on previous offenses are more likely to feel overly hostile (Bushman & Geen, 1990). Therefore, we hypothesized that anger rumination, which occurs when the subject of the rumination is an anger‐eliciting event (Sukhodolsky et al., 2001), might also result in hostile automatic thoughts. According to Wilkowski and Robinson's (2008) integrative cognitive model (ICM) of trait anger and reactive aggression, high trait anger levels make people more likely to see the ambiguous actions of others as hostile. This hostile attribution bias sets off more automatic rumination‐related processes and feeds anger and aggressive impulses. Because hostile attribution bias and hostile automatic thoughts both involve the cognition of hostility toward others, it is our view that hostile automatic thoughts can also trigger rumination (i.e., anger rumination). As a result, we propose that anger rumination and hostile automatic thoughts are mutually predictive. In light of this, we hypothesized that anger rumination and hostile automatic thoughts can independently mediate the association between bullying victimization and aggression; meanwhile, they can also sequentially mediate that connection.

### The present study

By examining the mediating effects of both anger rumination and hostile automatic thoughts, the current study sought to investigate how bullying victimization was connected to aggression in a three‐wave longitudinal study of Chinese undergraduate students. We put forth the following hypotheses:

Hypothesis 1: Anger rumination might mediate the relationship between bullying victimization and aggression.

Hypothesis 2: Hostile automatic thoughts might mediate the relationship between bullying victimization and aggression.

Hypothesis 3: Anger rumination and hostile automatic thoughts could predict each other.

Hypothesis 4: Anger rumination and hostile automatic thoughts could sequentially mediate the relationship between bullying victimization and aggression.

## METHODS

### Participants and procedure

A three‐wave longitudinal study was carried out, with a 6‐month delay between Time 1 and Time 2, and a 1‐year interval between Time 2 and Time 3. We planned to vary the intervals in order to test the covariant relationships among the variables. Undergraduate students at four Chinese universities were surveyed in June 2021 (Time 1), December 2021 (Time 2), and December 2022 (Time 3). In total, 1006 undergraduate students were recruited for the present study at T1; the number of participants at T2 was 1001; and there were 809 reserved participants (retention rate = 80.42%; 74.80% female; *M*_age_ = 19.63 years, *SD* = 0.82 years) who completed all three waves of the study. Samples were lost primarily for temporary absence and incomplete responses. There were no significant differences between participants who persisted (*n* = 809) and those who left the study (*n* = 197) for bullying victimization (*t*(268.40) = 1.90, *p = *.06, Cohen's *d* = 0.23), hostile automatic thoughts (*t*(1004) = 1.89, *p = *.06, Cohen's *d* = 0.12), and aggression (*t*(318.04) = 1.01, *p = *.32, Cohen's *d* = 0.11). Only anger rumination (*t*(1004) = 3.67, *p* = .001, Cohen's *d* = 0.23) indicated a significant difference.

The Ethical Review Committee of the research project at the Faculty of Psychology, Guangxi Normal University, reviewed and approved the study protocol. A team of qualified research assistants conducted the survey. All participants provided their informed consent before beginning the research; they were made aware that their participation was optional, and that they might refuse or drop out at any time. Then, participants were asked to complete paper questionnaires regarding our research variables. They were compensated for their participation in this study by receiving gifts at the end of the study.

### Measures

#### Bullying victimization

We used the Forms of Bullying Scale‐Victimization (FBS‐V) subscale of the Forms of Bullying Scale (FBS; Shaw et al., 2013) to measure a person's experience of being bullied. There are 10 items in the FBS‐V. Every question was answered on a 5‐point scale ranging from *almost never* to *every few weeks or more often*. A higher score implies more bullying victimization. In previous studies, the internal consistency of the FBS was reliable (Semenyna & Vasey, 2017). Cronbach's α for T1–T3 in this study was .949, .971, and .974, respectively.

#### Anger rumination

To evaluate anger rumination, the Anger Rumination Scale (ARS; Sukhodolsky et al., 2001) was utilized. It consists of 19 items, each of which is rated on a 4‐point scale from *almost never* to *almost always*. The higher the score, the more anger rumination. The ARS demonstrated good internal consistency as well as construct validity in a Chinese sample (Wang et al., 2018). In this study, T1–T3 had respective Cronbach's αs of .953, .966, and .973.

#### Hostile automatic thoughts

The Hostile Automatic Thoughts Scale (HATS; Snyder et al., 1997) was used to assess the frequency of hostile automatic thoughts in a person. It consists of 30 questions on a five‐point scale, with answers to each question ranging from *none at all* to *all the time*. The more hostile automatic thoughts one has toward other people, the higher his or her score will be. The internal reliability of the HATS has previously been shown to be high (McDermott et al., 2017). Cronbach's α at T1–T3 in this research was .975, .979, and .982, respectively.

#### Aggression

The 29‐item Buss–Perry Aggression Questionnaire (BPAQ; Buss & Perry, 1992) is used to measure aggression. Each item was scored using a 5‐point Likert scale from 1 (*extremely uncharacteristic of me*) to 5 (*extremely characteristic of me*). Higher scores indicate higher aggressive levels. The reliability and validity of the BPAQ have been shown to be high (Quan et al., 2019). At T1, T2, and T3 in this research, Cronbach's α was .928, .952, and .959, respectively.

#### Data analyses

For descriptive statistics and correlation analyses, SPSS Version 21.0 was utilized, while Mplus Version 8.0 was employed for confirmatory factor analysis (CFA) and structural equation modeling (SEM). First, we calculated descriptive statistics and correlations. Second, we used item parcels for our analyses because item parcels improve model fit and simplify the structure of the model (Little et al., 2002). For the three waves, all latent variables were randomly packed. Each latent variable consisted of four sets of items (three to four items per package), and the average item scores for each packet were calculated as observations. Third, we tested the measurement model using CFA (Anderson & Gerbing, 1988). Fourth, we tested a cross‐lagged model of anger rumination and hostile automatic thoughts at both T2 and T3. Finally, after controlling for gender, two structural models were tested using SEM. In Model 1, the serial path went from bullying victimization at T1 to anger rumination at T2, hostile automatic thoughts at T3, and aggression at T3. In Model 2, the serial path went from bullying victimization at T1 to hostile automatic thoughts at T2, anger rumination at T3, and aggression at T3. When the Tucker–Lewis index (TLI) and comparative fit index (CFI) are both greater than .90 and the root‐mean‐square error of approximation (RMSEA) and the standardized root‐mean‐square residual (SRMR) are both <.08, the model's goodness of fit is considered adequate (Hu & Bentler, 1999). To calculate the 95% confidence intervals (CIs) for the mediation's route, the bootstrap technique with 5000 resampled data was used. The significance level of the mediation effect was determined if the confidence interval did not contain 0.

## RESULTS

### Preliminary analyses

Table 1 presents the means, standard deviations, and correlations between the main variables. Significant positive correlations were found between bullying victimization, anger rumination, hostile automatic thoughts, and aggression at all three time points.

**TABLE 1:** Descriptive statistics and correlations between variables (*N* = 809).

	1	2	3	4	5	6	7	8	9	10	11	12
1. Bullying victimization (T1)	1											
2. Anger rumination (T1)	0.30***	1										
3. Hostile automatic thoughts (T1)	0.35***	0.56***	1									
4. Aggression (T1)	0.33***	0.51***	0.50***	1								
5. Bullying victimization (T2)	0.44***	0.19***	0.21***	0.20***	1							
6. Anger rumination (T2)	0.31***	0.60***	0.43***	0.40***	0.31***	1						
7. Hostile automatic thoughts (T2)	0.26***	0.38***	0.51***	0.40***	0.43***	0.53***	1					
8. Aggression (T2)	0.33***	0.41***	0.44***	0.53***	0.35***	0.53***	0.48***	1				
9. Bullying victimization (T3)	0.32***	0.22***	0.21***	0.22***	0.44***	0.30***	0.24***	0.31***	1			
10. Anger rumination (T3)	0.22***	0.43***	0.30***	0.37***	0.20***	0.57***	0.39***	0.40***	0.36***	1		
11. Hostile automatic thoughts (T3)	0.24***	0.30***	0.36***	0.36***	0.25***	0.41***	0.49***	0.38***	0.51***	0.55***	1	
12. Aggression (T3)	0.26***	0.33***	0.34***	0.47***	0.26***	0.42***	0.38***	0.54***	0.45***	0.57***	0.56***	1
Mean	0.27	1.94	1.86	2.05	0.26	1.84	1.75	2.05	0.31	1.81	1.75	2.07
Standard deviation	0.50	0.59	0.82	0.60	0.55	0.59	0.81	0.66	0.62	0.61	0.84	0.70

*** *p < *.001.

### Measurement model

The fitting of the measurement model at T1 (*χ* ^2^ = 312.82, *df* = 84, *χ* ^2^/*df* = 3.72, RMSEA = .05, CFI = .98, TLI = .98, SRMR = .04), T2 (*χ* ^2^ = 405.55, *df* = 84, *χ* ^2^/*df* = 4.83, RMSEA = .06, CFI = .98, TLI = .97, SRMR = .04), and T3 (*χ* ^2^ = 369.42, *df* = 84, *χ* ^2^/*df* = 4.40, RMSEA = .06, CFI = .98, TLI = .97, SRMR = .03) was acceptable.

### Cross‐lagged model

**FIGURE 1:**
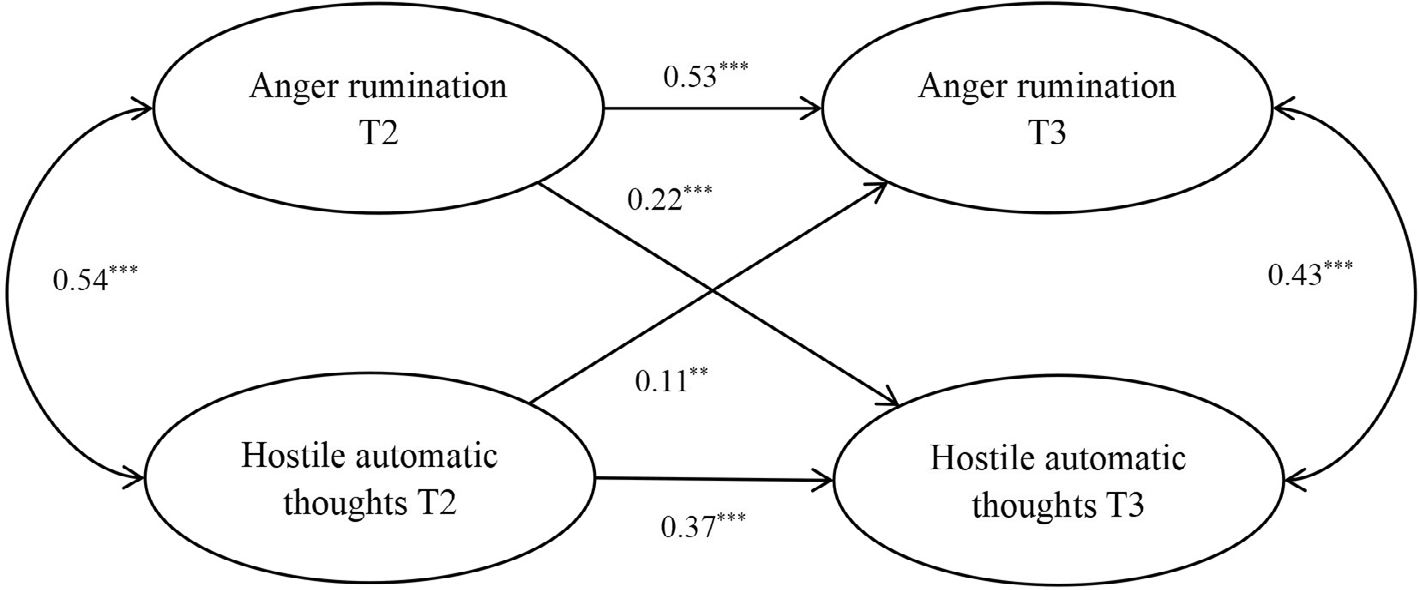
Cross‐lagged model of anger rumination and hostile automatic thoughts. Standardized regression coefficients are presented. ** *p < *.01, *** *p < *.001.

The fit indices of the cross‐lagged model of anger rumination and hostile automatic thoughts at T2 and T3 were acceptable: *χ* ^2^ = 267.34, *df* = 110, *χ* ^2^/*df* = 2.43, RMSEA = .04, CFI = .99, TLI = .99, SRMR = .01. Results showed that anger rumination at T2 significantly correlated with anger rumination at T3 (*β* = 0.53, *SE* = 0.04, *p < *.001) and hostile automatic thoughts at T3 (*β* = 0.22, *SE* = 0.04, *p < *.001). Hostile automatic thoughts at T2 were significantly associated with hostile automatic thoughts at T3 (*β* = 0.37, *SE* = 0.05, *p < *.001) and anger rumination at T3 (*β* = 0.11, *SE* = 0.04, *p < *.01). The results indicated that anger rumination and hostile automatic thoughts can predict each other longitudinally (see Figure 1).

### Longitudinal mediation model test

**FIGURE 2:**
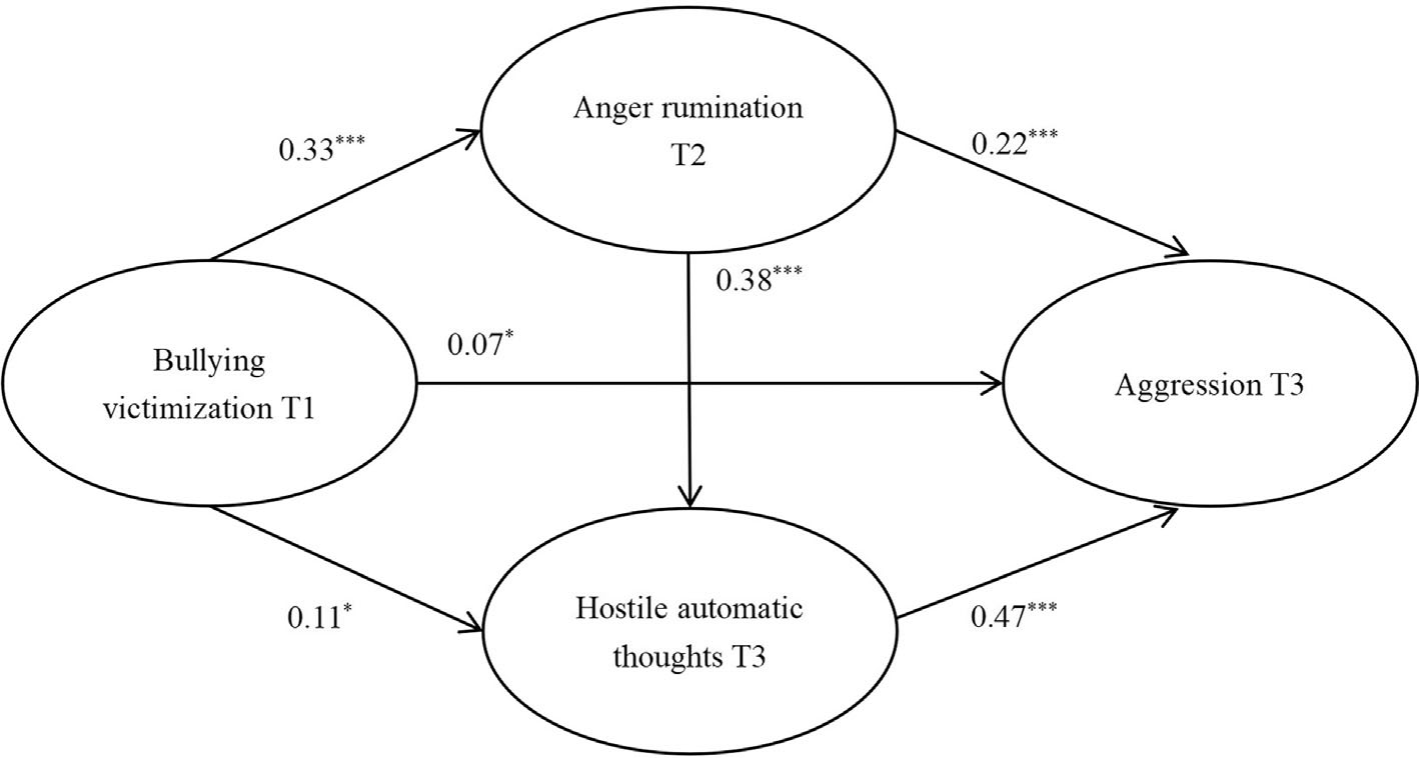
Model 1. The serial multiple mediation role of anger rumination and hostile automatic thoughts in the relationship between bullying victimization and aggression. Standardized regression coefficients are presented. * *p < *.05, *** *p < *.001.

The goodness‐of‐fit indices of Model 1 were acceptable: *χ* ^2^ = 428.29, *df* = 96, *χ* ^2^/*df* = 4.46, RMSEA = .07, SRMR = .03, CFI = .98, TLI = .98. Bullying victimization was associated with increased aggression at T3 (*β* = 0.07, *SE* = 0.03, *p < *.05), T2 anger rumination (*β* = 0.33, *SE* = 0.04, *p < *.001), and T3 hostile automatic thoughts (*β* = 0.11, *SE* = 0.04, *p < *.05). T2 anger rumination (*β* = 0.22, *SE* = 0.04, *p < *.001) and T3 hostile automatic thoughts (*β* = 0.47, *SE* = 0.03, *p < *.001) were both associated with T3 aggression. Furthermore, anger rumination at T2 was also positively correlated to hostile automatic thoughts at T3 (*β* = 0.38, *SE* = 0.04, *p < *.001; Figure 2). The results of the bootstrap estimation (see Table 2) showed that the total indirect effect of bullying victimization on aggression was significant (*β* = 0.18, *SE* = 0.03, *p < *.001, 95% CI [0.13, 0.23]). The indirect effects of T2 anger rumination (*β* = 0.07, *SE* = 0.02, *p < *.001, 95% CI [0.04, 0.11]) and T3 hostile automatic thoughts (*β* = 0.05, *SE* = 0.02, *p < *.05, 95% CI [0.01, 0.09]) were significant, indicating that these two variables could mediate the relationship between bullying victimization and aggression separately. In addition, the path of mediation of T1 bullying victimization, T2 anger rumination, T3 hostile automatic thoughts, and T3 aggression was also significant (*β* = 0.06, *SE* = 0.01, *p < *.001, 95% CI [0.04, 0.08]), supporting a serial mediation relationship.

**TABLE 2:** Total and indirect effects of Model 1.

Effect	*β*	*SE*	*p*	95% CIs	
				Lower	Upper
Direct effect	0.07	0.30	<0.05	0.02	0.14
Total indirect effect	0.18	0.03	<0.001	0.13	0.23
Bullying victimization T1 → Anger rumination T2 → Aggression T3	0.07	0.02	<0.001	0.04	0.11
Bullying victimization T1 → Hostile automatic thoughts T3 → Aggression T3	0.05	0.02	<0.05	0.01	0.09
Bullying victimization T1 → Anger rumination T2 → Hostile automatic thoughts T3 → Aggression T3	0.06	0.01	<0.001	0.04	0.08

*Note*: *β*, standardized regression coefficient.

**FIGURE 3:**
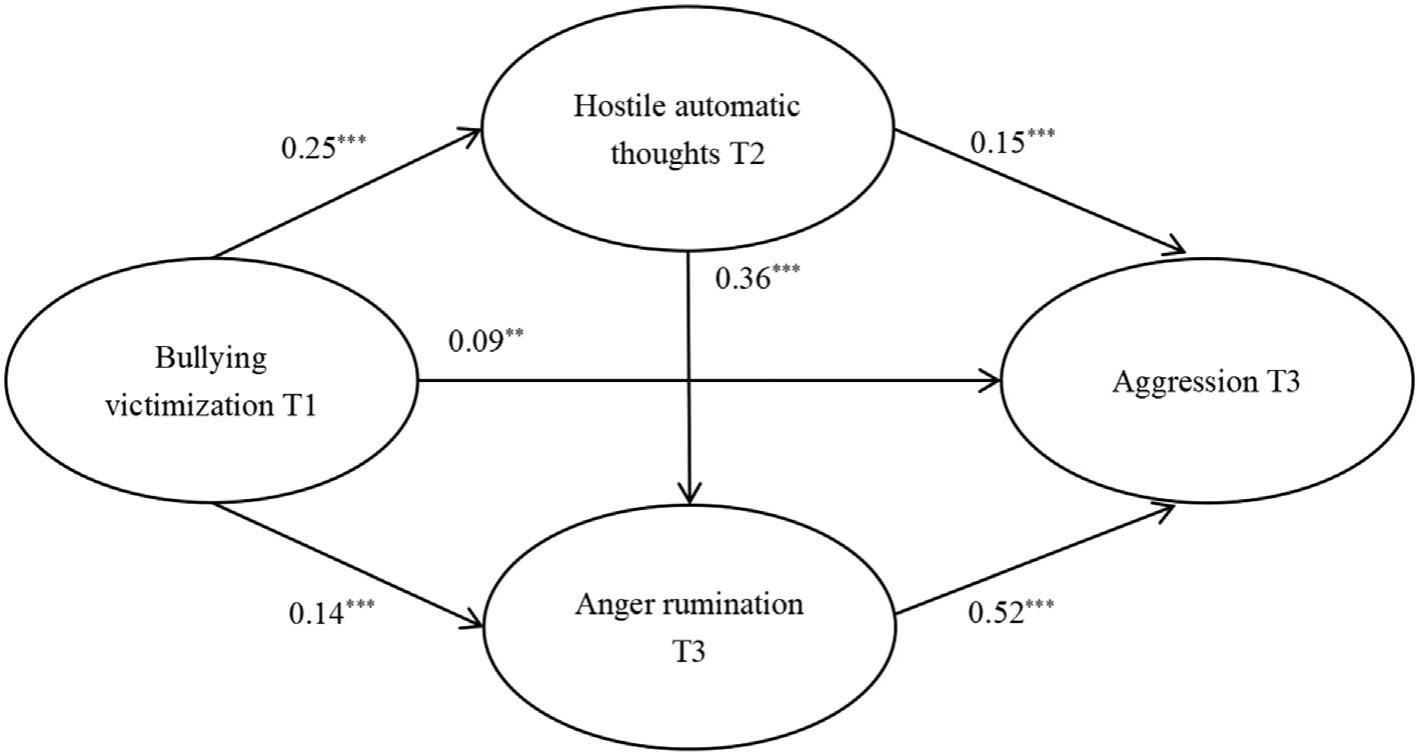
Model 2. The serial multiple mediation role of hostile automatic thoughts and anger rumination in the relationship between bullying victimization and aggression. Standardized regression coefficients are presented. ** *p < *.01, *** *p < *.001.

The goodness‐of‐fit indices of Model 2 were acceptable: *χ* ^2^ = 493.21, *df* = 96, *χ* ^2^/*df* = 5.14, RMSEA = .07, SRMR = .03, CFI = .98, TLI = .97. Results indicated that bullying victimization had a significantly positive effect on T2 hostile automatic thoughts (*β* = 0.25, *SE* = 0.04, *p < *.001), T3 anger rumination (*β* = 0.14, *SE* = 0.04, *p < *.001), and T3 aggression (*β* = 0.09, *SE* = 0.03, *p < *.01). T3 aggression was significantly predicted by T2 hostile automatic thoughts (*β* = 0.15, *SE* = 0.04, *p < *.001) and T3 anger rumination (*β* = 0.52, *SE* = 0.03, *p < *.001). In addition, hostile automatic thoughts at T2 positively correlated to anger rumination at T3 (*β* = 0.36, *SE* = 0.04, *p < *.001; Figure 3). The results of the bootstrap estimation (see Table 3) showed that the total indirect effect of bullying victimization on aggression was significant (*β* = 0.16, *SE* = 0.02, *p < *.001, 95% CI [0.12, 0.20]). The indirect effects of T2 hostile automatic thoughts (*β* = 0.04, *SE* = 0.01, *p < *.001, 95% CI [0.02, 0.07]) and T3 anger rumination (*β* = 0.07, *SE* = 0.02, *p < *.001, 95% CI [0.03, 0.11]) were significant, indicating that these two variables could mediate the relationship between bullying victimization and aggression separately. In addition, the mediating pathway of T1 bullying victimization, T2 hostile automatic thoughts, T3 anger rumination, and T3 aggression was also significant (*β* = 0.05, *SE* = 0.01, *p < *.001, 95% CI [0.03, 0.07]), supporting a serial mediation relationship.

**TABLE 3:** Total and indirect effects of Model 2.

Effect	*β*	*SE*	*p*	95% CIs	
				Lower	Upper
Direct effect	0.09	0.03	<.01	0.03	0.15
Total indirect effect	0.16	0.02	<.001	0.12	0.20
Bullying victimization T1 → Hostile automatic thoughts T2 → Aggression T3	0.04	0.01	<.001	0.02	0.07
Bullying victimization T1 → Anger rumination T3 → Aggression T3	0.07	0.02	<.05	0.03	0.11
Bullying victimization T1 → Hostile automatic thoughts T2 → Anger rumination T3 → Aggression T3	0.05	0.01	<.001	0.03	0.07

*Note*: *β*, standardized regression coefficient.

## DISCUSSION

The present study used a sample of Chinese undergraduates to explore the long‐term effects of bullying victimization on aggression by examining the mediating roles of anger rumination and hostile automatic thoughts. Results revealed that anger rumination (T2, T3) and hostile automatic thoughts (T2, T3) were longitudinally predictive of one another; anger rumination (T2, T3) and hostile automatic thoughts (T2, T3) mediated the associations between T1 bullying victimization and T3 aggression separately; anger rumination at T2 and hostile automatic thoughts at T3 sequentially mediated the associations between T1 bullying victimization and T3 aggression. In addition, another mediation model with hostile automatic thoughts at T2 as the first mediator and anger rumination at T3 as the second mediator was also of interest. The results of this study extend current theories and literature regarding the mechanisms associated with bullying victimization and aggression and provide a foundation for future prevention or/and intervention of aggression among undergraduate students.

In line with Hypothesis 1, our results suggest that T1 bullying victimization and T3 aggression are related, and this relationship may be mediated by anger rumination (T2, T3). Our findings support previous findings that bullying victimization is an important indicator of anger rumination (Peets et al., 2022; W. Zhu et al., 2020), and rumination due to anger can result in aggression (Li et al., 2019; Salguero et al., 2020; Smith et al., 2016; Wang et al., 2020). Derived from the social‐ecological diathesis‐stress model (Swearer & Hymel, 2015) and the multiple‐systems model of anger rumination (Denson, 2013), this mediating effect may be due to the fact that repeated exposure to bullying impairs individuals' cognitive processes, leading them to unconsciously ruminate on anger‐related events. Those who tend to engage in anger rumination may have poor inhibitory control and difficulty disengaging their attention focus, and this reduced capacity for self‐control may increase the likelihood of an individual's aggressive behaviors. In other words, bullying victimization may lead individuals to be more likely to recall and reflect on prior anger‐eliciting incidents (i.e., rumination of being bullied by others), which may further lead to aggressive behaviors.

Consistent with Hypothesis 2, we discovered that T2 and T3 hostile automatic thoughts can also mediate the connection between T1 bullying victimization and T3 aggression. Prior research has suggested that negative experiences influence negative automatic thoughts (Flouri & Panourgia, 2014; Park et al., 2018; R. Zhang et al., 2022), especially experiences of peer victimization (Edalati et al., 2018). Furthermore, related research has established that hostile automatic thoughts are significant predictors of different kinds of aggressive behaviors (Balan et al., 2018; Schniering & Rapee, 2004). Our results provide further evidence that being the victim of bullying makes a person more likely to have hostile automatic thoughts, which encourage aggressive behaviors. One possible explanation, based on Beck's (1976) cognitive theory and the social‐ecological diathesis‐stress model (Swearer & Hymel, 2015), is that repeated exposure to uncontrollable stressful life events can have an effect on people's cognitive processes, leading them to view and interpret stressful events in a negative and hostile manner. Consequently, when faced with novel life stressors, individuals may automatically harbor hostile thoughts about themselves and thus react with aggressive behaviors.

In line with Hypothesis 3 and Hypothesis 4, we found that T2 anger rumination can predict T3 hostile automatic thinking across time, and conversely, T2 hostile automatic thinking can also predict T3 anger rumination 1 year later, with anger rumination and hostile automatic thoughts sequentially mediating the link between bullying victimization and aggression. In other words, T1 bullying victimization can influence T3 aggression not only through the path of anger rumination (T2) to hostile automatic thoughts (T3), but also through the path of hostile automatic thoughts (T2) to anger rumination (T3). The social‐ecological diathesis‐stress model (Swearer & Hymel, 2015) posits that persistent environmental stressors can influence the occurrence of aggression through the modification of an individual's internal cognition, while the SIP model (Crick & Dodge, 1994) proposes that the cognitive factors linked to aggression may be reciprocally influenced by the function of a shared aggressive schema. Combining the perspectives of these two theories, our results may therefore be explained by the fact that both anger rumination and hostile automatic thoughts may function as cognitive factors in the impact of bullying victimization on aggression, so that they can both activate aggressive schema stored in memory; through this shared aggressive schema, these two variables may facilitate the emergence and growth of one another, which would explain the mutually predictive relationship between these two variables and their joint effects on aggression.

This study has some research and practical implications. First, our research expanded understanding of the mechanisms through which bullying victimization promotes future aggression by identifying the linkages between anger rumination and hostile automatic thoughts and the influence of these processes on the start of aggression. This will be helpful for the advancement of more thorough models to comprehend aggression as well as for further in‐depth study in this field. Second, our findings suggest that interventions targeted at decreasing individuals' anger rumination and hostile automatic thoughts may help mitigate their aggression. Such practices as mindfulness (Spek et al., 2013) and cognitive behavioral therapy (Querstret & Cropley, 2013) have been shown to be effective in reducing anger rumination; and the cognitive bias modification program (Hawkins & Cougle, 2013), which is successful in lowering hostile interpretation bias and reactivity to anger in the face of interpersonal insult, can be used to intervene in hostile automatic thoughts.

There are limitations that might open up new research possibilities. For example, the subjects were restricted to Chinese undergraduates in our study. Given the prevalence of bullying victimization in both elementary and middle school adolescents, future studies could recruit larger samples from these populations to replicate the present findings for cross‐group generalizability. At the same time, causal inferences are difficult to draw because the current study relied exclusively on self‐reported data on variables collected over three time points. Experiments can be employed to explore causal relationships among these four variables in the future.

## CONCLUSION

Using a sample of Chinese undergraduate students, this study explored the long‐term impacts of bullying victimization on aggression by examining the mediating effects of anger rumination and hostile automatic thoughts. Findings from our study provide new insights into the current literature by demonstrating the multiple mediation roles of anger rumination and hostile automatic thoughts in the connection between bullying victimization and aggression. Therefore, the negative consequences of bullying victimization and its impact on aggressive behaviors should be taken into account when establishing targeted treatments aimed at reducing future aggressive behaviors in undergraduate students.

## CONFLICT OF INTEREST

The authors declare there are no conflicts of interest.

## ETHICS STATEMENT

The Ethical Review Committee of the research project at the Faculty of Psychology, Guangxi Normal University, reviewed and approved the study protocol. All participants provided their informed consent before beginning the research.
